# Management and follow-up of gallbladder polyps: updated joint guidelines between the ESGAR, EAES, EFISDS and ESGE

**DOI:** 10.1007/s00330-021-08384-w

**Published:** 2021-12-17

**Authors:** Kieran G. Foley, Max J. Lahaye, Ruedi F. Thoeni, Marek Soltes, Catherine Dewhurst, Sorin Traian Barbu, Yogesh K. Vashist, Søren Rafael Rafaelsen, Marianna Arvanitakis, Julie Perinel, Rebecca Wiles, Stuart Ashley Roberts

**Affiliations:** 1 Department of Clinical Radiology, Royal Glamorgan Hospital, Llantrisant, UK; 2 Department of Radiology, Netherlands Cancer Institute, Amsterdam, The Netherlands; 3 Department of Radiology and Biomedical Imaging, University of California, San Francisco Medical School, San Francisco, CA USA; 4 1st Department of Surgery LF UPJS a UNLP, Kosice, Slovakia; 5 Department of Radiology, Mercy University Hospital, Grenville Place, Cork, Ireland; 6 4th Surgery Department, University of Medicine and Pharmacy “Iuliu Hatieganu”, Cluj-Napoca, Romania; 7 Clinics of Surgery, Department General, Visceral and Thoracic Surgery, Asklepios Goslar, Germany; 8 Department of Radiology, Clinical Cancer Centre, Vejle Hospital, University of Southern Denmark, Odense M, Denmark; 9 Department of Gastroenterology, Erasme University Hospital ULB, Brussels, Belgium; 10 Department of Hepatobiliary and Pancreatic Surgery, Edouard Herriot Hospital, Lyon, France; 11 Department of Radiology, Liverpool University Hospitals NHS Foundation Trust, Liverpool, UK; 12 Department of Radiology, University Hospital of Wales, Cardiff, UK

**Keywords:** Gallbladder, Polyps, Neoplasms, Ultrasonography, Cholecystectomy

## Abstract

**Abstract:**

**Main recommendations:**

1. Primary investigation of polypoid lesions of the gallbladder should be with abdominal ultrasound. Routine use of other imaging modalities is not recommended presently, but further research is needed. In centres with appropriate expertise and resources, alternative imaging modalities (such as contrast-enhanced and endoscopic ultrasound) may be useful to aid decision-making in difficult cases. Strong recommendation, low–moderate quality evidence.
2. Cholecystectomy is recommended in patients with polypoid lesions of the gallbladder measuring 10 mm or more, providing the patient is fit for, and accepts, surgery. Multidisciplinary discussion may be employed to assess perceived individual risk of malignancy. Strong recommendation, low-quality evidence.
3. Cholecystectomy is suggested for patients with a polypoid lesion and symptoms potentially attributable to the gallbladder if no alternative cause for the patient’s symptoms is demonstrated and the patient is fit for, and accepts, surgery. The patient should be counselled regarding the benefit of cholecystectomy versus the risk of persistent symptoms. Strong recommendation, low-quality evidence.
4. If the patient has a 6–9 mm polypoid lesion of the gallbladder and one or more risk factors for malignancy, cholecystectomy is recommended if the patient is fit for, and accepts, surgery. These risk factors are as follows: age more than 60 years, history of primary sclerosing cholangitis (PSC), Asian ethnicity, sessile polypoid lesion (including focal gallbladder wall thickening > 4 mm). Strong recommendation, low–moderate quality evidence.
5. If the patient has either no risk factors for malignancy and a gallbladder polypoid lesion of 6–9 mm, or risk factors for malignancy and a gallbladder polypoid lesion 5 mm or less, follow-up ultrasound of the gallbladder is recommended at 6 months, 1 year and 2 years. Follow-up should be discontinued after 2 years in the absence of growth. Moderate strength recommendation, moderate-quality evidence.
6. If the patient has no risk factors for malignancy, and a gallbladder polypoid lesion of 5 mm or less, follow-up is not required. Strong recommendation, moderate-quality evidence.
7. If during follow-up the gallbladder polypoid lesion grows to 10 mm, then cholecystectomy is advised. If the polypoid lesion grows by 2 mm or more within the 2-year follow-up period, then the current size of the polypoid lesion should be considered along with patient risk factors. Multidisciplinary discussion may be employed to decide whether continuation of monitoring, or cholecystectomy, is necessary. Moderate strength recommendation, moderate-quality evidence.
8. If during follow-up the gallbladder polypoid lesion disappears, then monitoring can be discontinued. Strong recommendation, moderate-quality evidence.

**Source and scope:**

These guidelines are an update of the 2017 recommendations developed between the European Society of Gastrointestinal and Abdominal Radiology (ESGAR), European Association for Endoscopic Surgery and other Interventional Techniques (EAES), International Society of Digestive Surgery–European Federation (EFISDS) and European Society of Gastrointestinal Endoscopy (ESGE). A targeted literature search was performed to discover recent evidence concerning the management and follow-up of gallbladder polyps. The changes within these updated guidelines were formulated after consideration of the latest evidence by a group of international experts. The Grading of Recommendations Assessment, Development and Evaluation (GRADE) system was adopted to define the strength of recommendations and the quality of evidence.

**Key Point:**

*• These recommendations update the 2017 European guidelines regarding the management and follow-up of gallbladder polyps.*

## Introduction

The management of gallbladder polyps remains a clinical dilemma. Gallbladder polyps are a common finding during trans-abdominal ultrasound (TAUS) in adults, yet gallbladder cancer is a relatively infrequent diagnosis [1]. Despite this, detection of malignancy at an early stage of disease is critical to improve survival rates because gallbladder cancer is associated with a dismal prognosis [2]. In 2017, original joint-societal guidelines concerning the management and follow-up of gallbladder polyps were published between the European Society of Gastrointestinal and Abdominal Radiology (ESGAR), European Association for Endoscopic Surgery and other Interventional Techniques (EAES), International Society of Digestive Surgery—European Federation (EFISDS) and European Society of Gastrointestinal Endoscopy (ESGE) [3]. The group originally planned an update and stated that the guidelines should not be a barrier to further research, which was greatly needed because of the limited evidence base. Here, we update the joint European guidelines by incorporating new evidence regarding the management of gallbladder polyps into its recommendations.

## Methods

Contributors to the original guidelines (C.D., M.L., S.R., R.T. from ESGAR; M.S. from EAES; S.B., J.P., Y.V. from EFISDS; M.A. from ESGE) were contacted in May 2020 (by R.W. and S.A.R.—previous guidelines Chairs) to ascertain their interest in participating toward these guideline revisions. All responded positively and agreed to contribute further, allowing continuation of expert knowledge to be updated with the latest evidence. The ESGAR guidelines committee appointed a new Chair (K.F.) to facilitate the guideline revision.

A literature search was performed on July 9, 2020, to update the previous evidence that was considered in the original guidelines. The search strategy was designed in Medline using the OVID platform (Supplementary Material). The abstracts of potentially relevant articles from 2015 onwards were considered (K.F.). A list of articles with relevance to gallbladder polyps was compiled and distributed to the group.

After the scope of the guidelines were re-visited, the original statements from the 2017 guidelines were re-shared with the group to evaluate their current clinical applicability. Again, consensus was determined by a series of Delphi questionnaires devised by the group Chair. A 5-point Likert scale was used to score each statement, where 1 = strongly disagree and 5 = strongly agree. Consensus was reached if at least seven out the nine contributors (77.8%) scored the statement as 4 or 5. The first Delphi questionnaire re-scored the original statements but none of the original statements were kept unchanged. Updated statements were drafted by the group Chair based on the evidence obtained from the literature review. All group members considered each new statement independently and scored their agreement blinded to others in the group. Group members were asked to consider both the updated and original evidence used to develop the 2017 guidelines [3]. Further Delphi rounds were performed when consensus was not reached for at least one statement and the relevant statement was re-drafted and re-distributed by the Chair. In total, three Delphi rounds were required to reach consensus on all guideline statements (hereafter called recommendations).

The guideline revision process followed the ESGAR recommendations for guideline development [4] and the principles of the Appraisal of Guidelines, Research and Evaluation (AGREE) II instrument [5]. Contributors were asked to list each article that they considered relevant to each recommendation, then independently graded the overall level of evidence using the Grading of Recommendations Assessment, Development and Evaluation (GRADE) system [6] for each relevant article. This information was returned to the group Chair along with the score for each statement. Formal comparison of GRADE classification between contributors was not performed. Any considerable discrepancies in GRADE classification was planned to be fed back to individual contributors by the Chair; however, this was not required. A draft manuscript was distributed amongst the group by the Chair for agreement. The final manuscript was approved by the ESGAR guidelines committee prior to submission for publication.

## Guideline recommendations

**Fig. 1:**
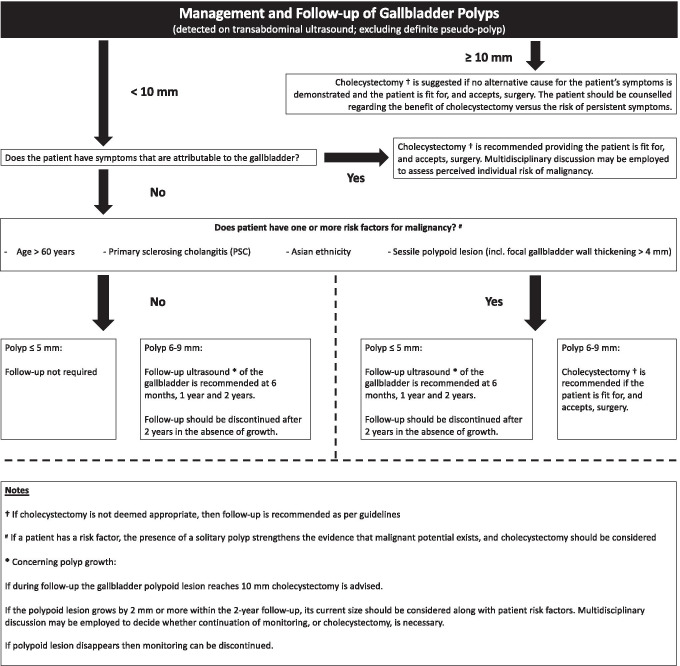
Management algorithm

A summary of the revised recommendations is provided within a management algorithm in Fig. 1. The recommendations below are based on the use of TAUS. Recommendations 1 to 7 have changed from the previous guidance, whereas recommendation 8 is unchanged. In cases of multiple polyps, the measurement of the largest polyp should be recorded and used to decide subsequent management.

**Fig. 2:**
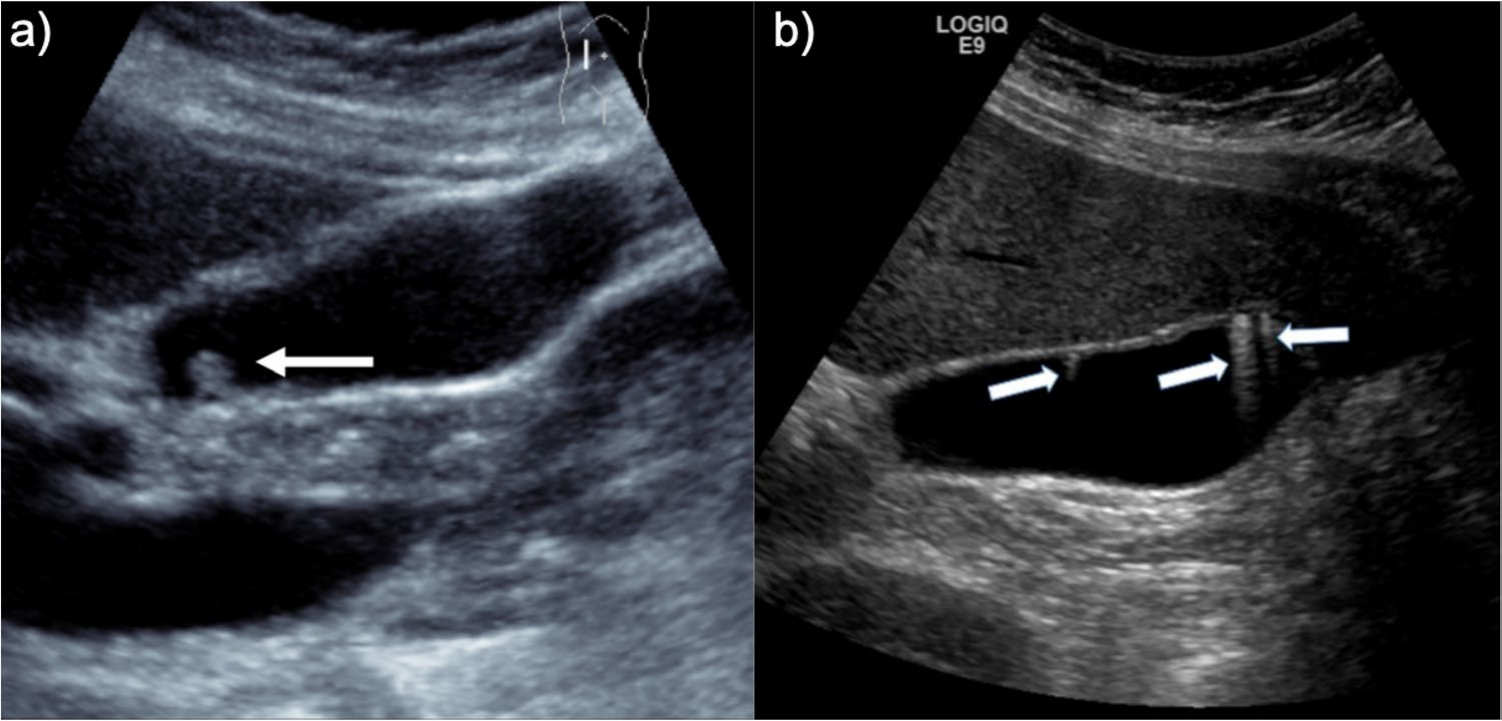
Selected images from two different patients show **a** a true gallbladder polyp and **b** a pseudo-polyp demonstrating posterior reverberation or ‘comet-tail’ artefact

As reported in the original guidelines, a gallbladder polyp is defined as an elevation of the gallbladder wall that protrudes into the gallbladder lumen (Fig. 2). The polyp should not be mobile or demonstrate posterior acoustic shadowing, features that are consistent with a calculus. A polyp can be sessile or pedunculated. If clear reverberation or ‘comet tail artefact’ is present, the lesion should be described as a pseudo-polyp (focal adenomyomatosis or a cholesterol polyp), in which case these guidelines do not apply. Again, it must be noted that not all pseudo-polyps demonstrate these ultrasound findings. An infiltrating or large mass should be considered as a gallbladder cancer, rather than a polyp.

Percentage agreement between contributors and GRADE of evidence are provided for each recommendation below. The explanatory text for each recommendation below summarises the literature published since 2015.

### Radiological investigation of gallbladder polypoid lesions

**Recommendation**

**Primary investigation of polypoid lesions of the gallbladder should be with abdominal ultrasound. Routine use of other imaging modalities is not recommended presently, but further research is needed. In centres with appropriate expertise and resources, alternative imaging modalities (such as contrast-enhanced and endoscopic ultrasound) may be useful to aid decision-making in difficult cases.**

**(Strong recommendation, low–moderate quality evidence, 100% agreement)**

TAUS remains the recommended primary imaging modality for the diagnosis and follow-up of gallbladder polyps, though several diagnostic accuracy studies conducted since 2015 have highlighted high false positive rates associated with this modality. The low positive predictive value (PPV) therefore has implications for increased cholecystectomy rates. However, the diagnostic accuracy should be considered in the context of low gallbladder polyp prevalence.

Martin et al. [7] conducted a systematic review which included 14 studies and 15,497 patients. In total, 1,259 had a gallbladder polyp. TAUS had a high false-positive rate (85.1%) for the diagnosis of gallbladder polyps when compared with pathological findings. Pickering et al. [8] conducted a retrospective study including 134 patients from four centres. Pseudo-polyps were found in 75 (55.9%) gallbladder specimens. Dysplastic or malignant polyps were seen in only six (4.5%) specimens and the PPV of TAUS for detecting neoplastic polyps was 4.5%. Spaziani et al. [9] conducted a single-centre, retrospective study including 2,631 patients who underwent cholecystectomy, of which 38 (1.4%) were diagnosed with gallbladder polyps on TAUS. False positives were found in 8 of those 38 patients (21.1%). A study by Lodhi et al. [10] demonstrated a PPV of 2.7%. Similar studies by Li et al. [11] (*n* = 2,290) and Metman et al. [12] (*n* = 108) found false positive rates of 1,661/2,290 (72.5%) and 62/65 (95.4%), respectively. These studies all suggested that surgical decisions should not be based on the TAUS findings alone, and that a more personalised approach be adopted.

Since the original guidelines, several studies have explored the potential of alternative modalities for detecting gallbladder polyps and differentiating dysplastic/malignant from benign polyps.

#### High-resolution ultrasound

Kim et al. [13] compared high-resolution ultrasound versus TAUS in a prospective single-centre study of 110 patients. Thirty-seven patients had cancer (33.6%), and 73 had polyps (66.4%). High-resolution features of neoplastic polyps included a single lobular surface, vascular core, hypoechoic polyp and hypoechoic foci. However, a polyp size of greater than 1 cm remained independently associated with a neoplastic polyp (odds ratio = 7.5, *p* = 0.02), resulting in a sensitivity and specificity of 66.7% and 89.1%, respectively.

#### Endoscopic ultrasound

A Cochrane systematic review by Wennmacker et al. comparing TAUS with endoscopic ultrasound (EUS) was published in 2018 [14]. Indirect comparison was only possible because limited numbers of patients received both tests, meaning meta-analysis could not be performed. Three studies (*n* = 209) investigating EUS to differentiate true and pseudo-polyps were analysed. The sensitivity of EUS was 0.85 (95% confidence intervals (CI) 0.46 to 0.97) and the specificity was 0.90 (95% CI 0.78 to 0.96) compared to a sensitivity of 0.68 (95% CI 0.44 to 0.85) and specificity of 0.79 (95% CI 0.57 to 0.91) in six studies (*n* = 1078) investigating TAUS.

Three studies (*n* = 351) investigating EUS to differentiate dysplastic polyps and non-dysplastic polyps found the sensitivity of EUS was 0.86 (95% CI 0.76 to 0.92) and the specificity was 0.92 (95% CI 0.85 to 0.95). This was compared to a sensitivity of 0.79 (95% CI 0.62 to 0.90) and the specificity of 0.89 (95% CI 0.68 to 0.97) with TAUS in four studies (*n* = 1,009). The review concluded that insufficient evidence exists to show that EUS is better than TAUS. No studies investigated EUS for the detection of gallbladder polyps.

#### Contrast-enhanced ultrasound

There has been a growing interest in contrast-enhanced ultrasound (CEUS) to improve the diagnosis and risk stratification of gallbladder polyps [15–21]. These are mostly small, single-centre studies with selected cohorts of patients.

Notable examples include a study by Zhang et al. [15] which recruited 105 patients with gallbladder lesions. Seventeen patients had cancer, and 88 were benign. The sensitivity, specificity, PPV, negative predictive value (NPV) and accuracy of CEUS were 94.1%, 95.5%, 80.0%, 98.8% and 95.2%, respectively. These were significantly higher than conventional ultrasound (82.4%, 89.8%, 60.9%, 96.3% and 88.6%, respectively).

Fei et al. [17] attempted to differentiate adenoma from cholesterol polyps in a prospective single-centre including 112 consecutive patients. There were differences in patient age, lesion size, echogenicity, stalk width, enhancement intensity and vascularity of lesion between the two groups. Multiple logistic regression analysis showed that enhancement intensity, stalk of lesion and vascularity were independent factors associated with adenoma.

Dong et al. [18] conducted a prospective single-centre study recruiting 59 patients with focal gallbladder lesions, including 15 with adenocarcinoma, and 29 with polyps. CEUS features of arterial-phase irregular intralesional vascularity (10/15, 66.7%), late-phase hypo-enhancement (12/15, 80.0%), destruction of gallbladder wall (8/15, 53.3%) and infiltration to the adjacent liver (6/15, 40.0%) were significantly higher in gallbladder malignancy. The sensitivity, specificity and accuracy of CEUS were 93.3%, 88.5% and 100%, respectively.

#### Magnetic resonance imaging

A retrospective, single-centre study by Kitazume et al. [22] which included 91 patients (13 malignant, 78 benign) compared diffusion-weighted imaging (DWI) to three morphological features (mass, disrupted mucosal line and absence of two-layered pattern). When two or more morphological features were positive for malignancy, the sensitivity, specificity and accuracy were 76.9%, 84.0% and 83.0%, respectively. When morphological features were combined with apparent diffusion coefficient (ADC) values of less than 1.2 × 10^−3^ mm^2^/s, or a lesion to spinal cord ratio of more than 0.48, the sensitivity, specificity and accuracy were 73.0%, 96.2% and 92.9%, respectively.

#### Positron emission tomography

One small, single-centre study (*n* = 30, with 12 malignancies) investigated ^18^fluorine-fluorodeoxyglucose (18F-FDG) positron emission tomography (PET) to differentiate benign and malignant gallbladder wall thickening [23]. Using a threshold of 8.5 mm, the sensitivity and specificity of detecting malignancy was 94% and 67%. The mean standardised uptake value (SUV) uptake was 7.5 (benign = 4.5, malignant = 14.3, *p* = 0.01). Using a SUV threshold of 5.95, the sensitivity and specificity of detecting malignancy was 92% and 79%. Overall, the sensitivity, specificity, PPV, NPV and diagnostic accuracy of FDG-PET was 91%, 79%, 77%, 92% and 84%, respectively.

#### Radiomics

A few studies have investigated radiomics to differentiate benign and malignant gallbladder polyps [24, 25]. These were small, single-centre studies that used quantitative imaging data in attempt to improve the performance of conventional diagnostic techniques. These studies reported variable results, with potentially significant metrics demonstrated, but the studies are limited by their sample size and methodology.

The group acknowledge that although these studies investigating alternative modalities are promising, further research is needed to reliably improve the accurate differentiation of benign from malignant gallbladder polyps. Most studies are of limited value due to their small sample size and study design. To make these guidelines useful for all radiology departments, TAUS continues to be recommended. TAUS is a highly repeatable and reproducible technique [26], an essential criterion when monitoring an abnormality over time. Centres with sufficient resources and expertise may find alternative modalities useful, particularly in patients considered high risk for cholecystectomy.

### Cholecystectomy for polypoid lesions of the gallbladder

**Recommendation**

**Cholecystectomy is recommended in patients with polypoid lesions of the gallbladder measuring 10 mm or more, providing the patient is fit for, and accepts, surgery. Multidisciplinary discussion may be employed to assess perceived individual risk of malignancy.**

**(Strong recommendation, low-quality evidence, 100% agreement)**

Polyp size remains an independent risk factor for malignancy. One large observational study by Wennmacker et al. [27] suggested that the 10 mm threshold alone may not be a sufficient indication to perform cholecystectomy. The authors studied a national cohort of histopathologically proven gallbladder polyps to distinguish neoplastic from non-neoplastic polyps between 2003 and 2013. In total, 2,085 of 220,612 cholecystectomies contained a polyp (0.9%). Of these, 56.4% were neoplastic (40.1% premalignant, 59.9% malignant) and 43.6% non-neoplastic (41.5% cholesterol polyp, 37.0% adenomyomatosis, 21.5% other). Pathological polyp size was reported in 1,059 patients. There was a significant difference in size between neoplastic and non-neoplastic polyps (18.1 mm vs 7.5 mm, *p* < 0.001). Fifty percent of all polyps were 10 mm or greater. Using this size threshold, the sensitivity, specificity, PPV and NPV for neoplastic polyps were 68.1%, 70.2%, 72.9% and 65.1%, respectively.

Several studies [25, 28–34] have investigated alternative size thresholds at which to intervene, but the overall quality of evidence remains low. These include thresholds of 12 mm [28, 29], 13 mm [30, 31], 14 mm [25] and 15 mm [32–34]. In the absence of an alternative size threshold with better quality evidence, the group chose to leave the 10 mm threshold unchanged. Multidisciplinary team (MDT) meetings may be used to discuss the best management of individual patients.

### Polypoid lesion of the gallbladder with symptoms potentially attributable to the gallbladder

**Recommendation**

**Cholecystectomy is suggested if no alternative cause for the patient’s symptoms is demonstrated and the patient is fit for, and accepts, surgery. The patient should be counselled about the benefit of cholecystectomy versus the risk of persistent symptoms.**

**(Strong recommendation, low-quality evidence, 100% agreement)**

Again, there was limited and low-quality evidence regarding the value of cholecystectomy in patients with symptoms potentially attributable to the gallbladder. Polyps themselves are unlikely to cause pain; however, some studies have reported associations between polyp formation [11], adenoma [35] and malignancy [36, 37] with gallstones and/or cholecystitis. There were concerns of reports that symptoms can persist following cholecystectomy. One such study reported that 85 of 140 symptomatic patients (60.7%) reported ongoing pain after their cholecystectomy [38]. Hence, the group emphasise that symptomatic patients with gallbladder polyps should be counselled about the potential benefits of cholecystectomy versus the risks of persistent pain.

### Risk factors for malignancy in patients with polypoid lesions of the gallbladder measuring 6–9 mm

**Recommendation**

**If the patient has a 6–9 mm polypoid lesion of the gallbladder and one or more risk factors for malignancy, cholecystectomy is recommended if the patient is fit for, and accepts, surgery. These risk factors are:**

- **Age more than 60 years**
- **History of primary sclerosing cholangitis (PSC)**
- **Asian ethnicity**
- **Sessile polypoid lesion (including focal gallbladder wall thickening > 4 mm)**

**(Strong recommendation, low–moderate quality evidence, 100% agreement)**

#### Patient age

Age continues to be a prognostic factor for gallbladder cancer. Much like polyp size, optimum age thresholds defining the risk factor are variable between studies and in many cases are entirely arbitrary. The best available evidence is a systematic review published by Elmasry et al. in 2016 [39], which included 12 studies and 5,482 gallbladder polyps. The review identified an age threshold of more than 60 years as a significant risk factor for malignancy. Given the ageing population worldwide, and an incidence of malignant polyps of just 0.6% in this review, the group suggested increasing the age threshold to more than 60 years. However, we note that the available evidence is of moderate quality at best.

#### Primary sclerosing cholangitis

Few studies related to primary sclerosing cholangitis (PSC) as a risk factor in gallbladder polyps have been published since the original guidelines were developed. van Erp et al. [40] studied 453 patients with PSC across two centres. Gallbladder polyps were discovered in 16%. The gallbladder cancer rate was 8.8 (95% CI 1.8–25.7) per 1000 person-years. Another study by Sagvand et al. [41] found polyps in 10.6% of patients with PSC (*n* = 363). Two cases of adenocarcinoma were found in 4 mm and 7 mm polyps, the remaining four of six adenocarcinomas measured > 10 mm. PSC is still considered a risk factor, because even some smaller polyps appear to have malignant potential in this population.

#### Asian ethnicity

Again, there were few recent studies examining ethnicity as a risk factor for malignancy. The systematic review by Elmasry et al. [39] provided more evidence that Indian ethnicity was associated with increased risk of malignancy. Another systematic review by Babu et al. [42] which included 43 articles and 11,685 patients with polyps found that the risk of malignancy was higher in Asian populations and suggested increased duration of surveillance in these populations. The group decided that, based on the latter systematic review, this risk factor should be broadly extended to include all Asian populations.

#### Sessile polyp (including focal gallbladder wall thickening > 4 mm)

Several observational studies have identified sessile polyps as a risk factor for malignancy in multi-variable analysis [25, 30, 32, 43]. Notably, Terzioglu et al. [43] studied 278 patients (5% had neoplastic polyps) in a single-centre study and reported that sessile morphology (*p* < 0.001) was independent predictor of a neoplastic polyp. The group elected to continue recommending that a sessile polyp, including focal gallbladder wall thickening > 4 mm, is considered a risk factor. An important consideration is that true wall thickening should be differentiated from adenomyomatosis [44].

Several studies identified in the literature review also reported a solitary polyp as a risk factor for malignancy [25, 27, 30, 32, 39, 43]. However, details on the size of the polyp, and the association with other risk factors such as age, were often lacking. To ensure these revised guidelines are pragmatic and easily adoptable, the group decided not to add a solitary polyp to the list of risk factors above. However, the group would like to emphasise that when a patient has one of the four risk factors, the presence of a solitary polyp strengthens the evidence that malignant potential exists, and that cholecystectomy should be considered.

### Monitoring of small polypoid lesions in patients with risk factors, or in patients without risk factors

**Recommendation**

**If the patient has either:**

- **No risk factors for malignancy and a gallbladder polypoid lesion of 6–9 mm or**
- **Risk factors for malignancy and a gallbladder polypoid lesion 5 mm or less**

**Follow-up ultrasound of the gallbladder is recommended at 6 months, 1 year and 2 years. Follow-up should be discontinued after 2 years in the absence of growth.**

**(Moderate strength recommendation, moderate-quality evidence, 89% agreement)**

Recently, a large retrospective study by Szpakowski and Tucker reported outcomes of gallbladder polyps in a patient cohort monitored for 20 years from a population of more than 600,000 [45]. The unadjusted gallbladder cancer rate per 100,000 person-years was 11.3 (95% CI 6.2–16.3) and increased with greater polyp size, from 1.3 (95% CI 0.7–6.5) in polyps less than 6 mm, to 128.2 (95% CI 9.4–217.0) in polyps 10 mm or greater. Additionally, gallbladder cancer rates in this cohort study were similar in patients with and without polyps on initial ultrasound (0.053% vs 0.054%, respectively). The authors suggested that a limited strategy of one or two follow-up ultrasound investigations in the subsequent 1–2 years should provide adequate duration of monitoring. The group felt that the frequency and duration of monitoring recommended is sufficient to provide adequate reassurance for patients and clinicians, and wish to emphasise that monitoring should be reserved for those who are potentially fit for surgery.

### Monitoring of small polypoid lesions in patients without risk factors

**Recommendation**

**If the patient has no risk factors for malignancy, and a gallbladder polypoid lesion of 5 mm or less, follow-up is not required.**

**(Strong recommendation, moderate-quality evidence, 100% agreement)**

The group acknowledges that a few cases of gallbladder cancer in polyps measuring 5 mm or less have been reported in the literature to date, and predominately in those patients with risk factors. However, the new data reported by Szpakowski and Tucker [45] was felt to provide insufficient justification for their ongoing follow-up. Szpakowski and Tucker hypothesised that the resources required for yearly surveillance would be considerable. For example, in polyps smaller than 10 mm, it was hypothesised that 95,624 ultrasound examinations after the first year would be required to detect one gallbladder cancer (equalling a rate of 1.05 per 100,000).

Therefore, the group felt a pragmatic approach to small gallbladder polyps was necessary, and, as such, do not recommend that gallbladder polyps measuring 5 mm or less are monitored.

This strategy broadly aligns with the American College of Radiology [46] and the Canadian Association of Radiologists Incidental Findings Working Group [47], who recommend that polyps less than 7 mm do not require follow-up.

### Management of gallbladder polypoid lesions that grow

**Recommendation**

**If during follow-up the gallbladder polypoid lesion reaches 10 mm, then cholecystectomy is advised.**

**If the polypoid lesion grows by 2 mm or more within the 2-year follow-up period, then the current size of the polypoid lesion should be considered along with patient risk factors. Multidisciplinary discussion may be employed to decide whether continuation of monitoring, or cholecystectomy, is necessary.**

**(Moderate strength recommendation, moderate-quality evidence, 78% agreement)**

Szpakowski and Tucker [45] provide the best evidence to date that slow growth is part of the natural history of gallbladder polyps, having 5-year and 10-year follow-up data on 13,236 and 4,923 patients, respectively. Their study reported that the cumulative probability of a gallbladder polyp growing by 2 mm or more at 10 years was small, but uniformly progressive during the follow-up period, reaching 66.2% (95% CI, 62.3–70.0%) in polyps initially smaller than 6 mm and 52.9% (95% CI, 47.1–59.0%) in those initially sized 6–10 mm.

The risk of polyps reaching 10 mm also increased linearly over time, with smaller polyps taking a longer duration to reach that size. In addition, growth to 10 mm was not associated with increased risk of gallbladder cancer. In total, 507 gallbladder polyps (with at least 1 follow-up ultrasound) reached a size of 10 mm, of which 210 (41.4%) were initially smaller than 6 mm and 297 (58.6%) were sized between 6 and 10 mm. In the 1549 person-years of follow-up, no gallbladder cancer was diagnosed. Importantly, the risk of subsequent growth in those with previous stability of polyp size progressed in a similar relationship to those without the period of initial stability. The authors reported that one patient (< 0.1%) whose polyp was stable for 3 years subsequently developed gallbladder cancer.

### Monitoring polypoid lesions that disappear

**Recommendation**

**If during follow-up the gallbladder polypoid lesion disappears, then monitoring can be discontinued.**

**(Strong recommendation, moderate-quality evidence, 89% agreement)**

This recommendation is unchanged from the previous guidance. The group found no new evidence to the contrary. Despite studies demonstrating poor PPV for gallbladder polyp detection, the systematic reviews of Martin et al. [7] and Wennmacker et al. [14] suggest the sensitivity and NPV of TAUS is high enough, and clinically acceptable, to discontinue monitoring of a polyp should one disappear.

## Limitations

Again, a lack of randomised data concerning the management of gallbladder polyps exists. It may not be feasible to conduct a randomised trial of monitoring strategies given the infrequent event rate of gallbladder cancer and costs associated with the size of study needed to power the clinical endpoints. Systematic reviews and large observational studies described above have been published since the previous version of the guidelines in attempt to discover important answers to these challenging clinical questions and have allowed these recommendations to be revised accordingly. Large, longitudinal European polyp registries may provide sufficient data to optimise patient management in the future. The group encourages further research in this area.

## Consideration of health economics

One further cost-effectiveness study has been published since Cairns et al. [48]. Patel et al. [49] have suggested that compliance with the original polyp monitoring guidelines may be cost-effective. Adherence to the European joint society guidelines could result in an estimated annual saving of £209,163 per 1,000 gallbladder polyps surveyed in the National Health Service (NHS), and result in an additional 12.5% of patients requiring cholecystectomy. However, compliance with guidelines was found to be poor. The data published by Szpakowski and Tucker since this study is likely to affect health economic models considerably. Further health economic research is needed to evaluate the cost-effectiveness of monitoring gallbladder polyps, particularly those measuring less than 10 mm.

## Compliance to guidelines

As Patel et al. [49] and other groups [50] have demonstrated, compliance with the previous version of the guidelines was poor. The primary reason was reported to be the considerable resources needed to complete the recommended follow-up schedule. The group hopes that there will be widespread adoption of the new recommendations, which no longer require follow-up for polypoid lesions 5 mm or less and suggest shorter follow-up duration, amongst all radiology departments and referring clinicians across Europe.

## Patient involvement

As stated in the previous guidelines, the group is aware that acceptance of these guidelines is likely to vary across Europe. The group is still not aware of any specific European focus groups. However, we recognise how important patient involvement is when developing and updating guidelines, and strongly advocate the inclusion of patient representatives when designing and conducting research.

## Updating the guidelines

These guidelines should be updated in or before 2025 if new evidence of good quality becomes available that warrant significant modification to these recommendations.

## Abbreviations

ADC: Apparent diffusion coefficient
AGREE: Appraisal of Guidelines, Research and Evaluation
CEUS: Contrast-enhanced ultrasound
CI: Confidence intervals
EAES: European Association for Endoscopic Surgery and other Interventional Techniques
EFISDS: International Society of Digestive Surgery -European Federation
ESGAR: European Society of Gastrointestinal and Abdominal Radiology
ESGE: European Society of Gastrointestinal Endoscopy
EUS: Endoscopic ultrasound
GRADE: Grading of Recommendations Assessment, Development and Evaluation
MRI: Magnetic resonance imaging
NPV: Negative predictive value
PET: Positron emission tomography
PPV: Positive predictive value
PSC: Primary sclerosing cholangitis
TAUS: Trans-abdominal ultrasound

## Declarations

### Guarantor

The scientific guarantor of this publication is Dr. Kieran Foley.

### Statistics and biometry

No complex statistical methods were necessary for this paper.

### Methodology

• Guideline development using Delphi consensus method.
